# Simulation of coronary fractional flow reserve and whole-cycle flow based on optical coherence tomography in individual patients with coronary artery disease

**DOI:** 10.1007/s10554-024-03151-6

**Published:** 2024-06-16

**Authors:** Niels Thue Olsen, Kaining Sheng

**Affiliations:** 1 Department of Cardiology, Copenhagen University Hospital - Herlev and Gentofte, Copenhagen, Denmark; 2 Department of Clinical Medicine, University of Copenhagen, Copenhagen, Denmark; 3 Department of Radiology, Copenhagen University Hospital - Rigshospitalet, Copenhagen, Denmark

**Keywords:** Coronary artery disease, Coronary physiology, Fractional flow reserve, Microvascular function, Computer simulation

## Abstract

Computer simulations of coronary fractional flow reserve (FFR) based on coronary imaging have emerged as an attractive alternative to invasive measurements. However, most methods are proprietary and employ non-physiological assumptions. Our aims were to develop and validate a physiologically realistic open-source simulation model for coronary flow, and to use this model to predict FFR based on intracoronary optical coherence tomography (OCT) data in individual patients. We included patients undergoing elective coronary angiography with angiographic borderline coronary stenosis. Invasive measurements of coronary hyperemic pressure and absolute flow and OCT imaging were performed. A computer model of coronary flow incorporating pulsatile flow and the effect of left ventricular contraction was developed and calibrated, and patient-specific flow simulation was performed. Forty-eight coronary arteries from 41 patients were included in the analysis. Average FFR was 0.79 ± 0.14, and 50% had FFR ≤ 0.80. Correlation between simulated and measured FFR was high (r = 0.83, p < 0.001). Average difference between simulated FFR and observed FFR in individual patients was − 0.009 ± 0.076. Overall diagnostic accuracy for simulated FFR ≤ 0.80 in predicting observed FFR ≤ 0.80 was 0.88 (0.75–0.95) with sensitivity 0.79 (0.58–0.93) and specificity 0.96 (0.79–1.00). The positive predictive value was 0.95 (0.75–1.00) and the negative predictive value was 0.82 (0.63–0.94). In conclusion, realistic simulations of whole-cycle coronary flow can be produced based on intracoronary OCT data with a new, computationally simple simulation model. Simulated FFR had moderate numerical agreement with observed FFR and a good diagnostic accuracy for predicting hemodynamic significance of coronary stenoses.

**Supplementary Information:**

The online version contains supplementary material available at 10.1007/s10554-024-03151-6.

## Introduction

Invasive measurement of fractional flow reserve (FFR) with a pressure-sensitive intracoronary wire is a well-established and guideline-recommended method to evaluate the hemodynamic significance of coronary stenosis [1–3]. Estimating FFR with computer simulations of coronary flow based on data from coronary imaging modalities has been shown to be feasible based on cardiac computed tomographic angiography [4, 5], invasive angiography [6–8], intravascular ultrasound [9–11], and optical coherence tomography (OCT) [12–16]. A simulation-based approach would allow simultaneous morphological and physiological assessment, could help planning for revascularization [17], and explicit modeling could expand our understanding of coronary physiology. However, many FFR-estimating methods are proprietary and most employ simplified assumptions of non-pulsatile flow, and myocardial-vascular interaction is seldomly included [18].

The aims of the current study were: (1) Develop a physiologic simulation model allowing patient-specific simulation of pulsatile whole-cycle coronary flow including aortic pressure variation, impact of epicardial coronary artery disease, side branch flow, and myocardial-vascular interaction in the coronary microcirculation, (2) Calibrate empirical components of the model against measured coronary flows and pressures, and (3) Test the accuracy of the simulation model in predicting measured FFR of individual coronary stenoses based on intracoronary OCT imaging data from patients with stable coronary disease.

## Materials and methods

### Transparency of methods and data

Project code has been made publicly available on GitHub [https://github.com/thueolsen/Coronary-simulation], including instructions on downloading JSim software for running the code. Anonymized data are available on reasonable request from the corresponding author.

### Patients

Participants were recruited among adult patients scheduled for routine elective invasive coronary angiography at our center with an established or suspected diagnosis of stable angina pectoris that had at least one coronary lesion with 40–90% diameter stenosis. Exclusion criteria included moderate or worse left-sided valvular disease, cardiomyopathy, left ventricular (LV) ejection fraction ≤ 30%, eGFR < 45 ml/min/1.73 m^2^, or contraindications to adenosine.

### Invasive procedure and coronary physiology

Invasive coronary angiography was performed with 6 French catheters from the radial artery in all patients. Intracoronary nitroglycerin was used before angiography, before physiology measurements, and before intravascular imaging. For intracoronary physiology measurements of resting full-cycle ratio, FFR, coronary flow reserve, and absolute hyperemic flow, we used a 0.014″ pressure and temperature sensitive coronary wire (PressureWire X, Abbott, Santa Clara, CA, USA) and a RayFlow perfusion microcatheter (Hexacath, Paris, France) with physiology measurements performed on CoroFlow software v3.01 (Coroventis Research AB, Uppsala, Sweden). Absolute flow during hyperemia was measured as reported elsewhere [19, 20].

### Intracoronary imaging with optical coherence tomography

OCT imaging was performed with a DragonFly Optis imaging catheter (Abbott, Santa Clara, CA, USA) connected to an OCT imaging console (Optis Mobile System, Abbott, Santa Clara, CA, USA). OCT imaging was performed using automated pull-back during intracoronary contrast administration, using survey-mode to image 75 mm of the proximal part of the coronary artery. In cases where lesions were more than 75 mm distal from the ostium, 2 overlapping pullbacks were performed.

### OCT image analysis

Image files were exported to an off-line OCT workstation (Ilumien Optis ORW, Abbott, Santa Clara, CA, USA). The lesion reference vessel size was measured by measuring the distance from the arterial medial layer in the longest and the shortest diameter in the least diseased segment of the artery immediately proximal to the stenosis. Reference vessel area (RVA) was calculated using the formula for an ellipse. Manual corrections of automated lumen detection were performed over the entire arterial pull-back. The smallest lumen area within the relevant part of the artery was recorded as the minimal lumen area (MLA). Plaque burden was calculated as 1-(MLA/RVA).

Text-files with comma-separated values containing data on the corrected lumen areas and minimal and maximal lumen diameters over the entire OCT-pullback length were exported from the OCT workstation to a spreadsheet (Excel, Microsoft, Redmond, WA, USA). Here, the lumen data were cropped to contain only the region of interest (from the proximal end of the artery to a position distal to the most diseased part of the artery). When data from overlapping pullbacks were merged within the spreadsheet, we used information on landmarks (especially prominent sidebranches) and their precise frame position from the manual review of the OCT-images. Data were now re-exported as csv-files to be used by the simulation software. OCT-measurements regarding reference vessel area and plaque burden were manually entered into the study database.

### Simulation model

A simulation model of was developed using JSim v. 2.21 [21]. JSim is a software platform for writing and running physiological simulation models provided free for non-commercial use by the National Simulation Resource at the University of Washington, Seattle, WA, USA. The full source code of our model has been made available online (see above). The model includes an inlet (pulsating aortic pressure), a stenotic epicardial coronary vessel (the region of interest, ROI), idealized side-branch flow, the distal microcirculation, a left ventricular cavity with time-varying pressure impacting the microcirculation, and an outlet (right atrial pressure).

**Fig. 1:**
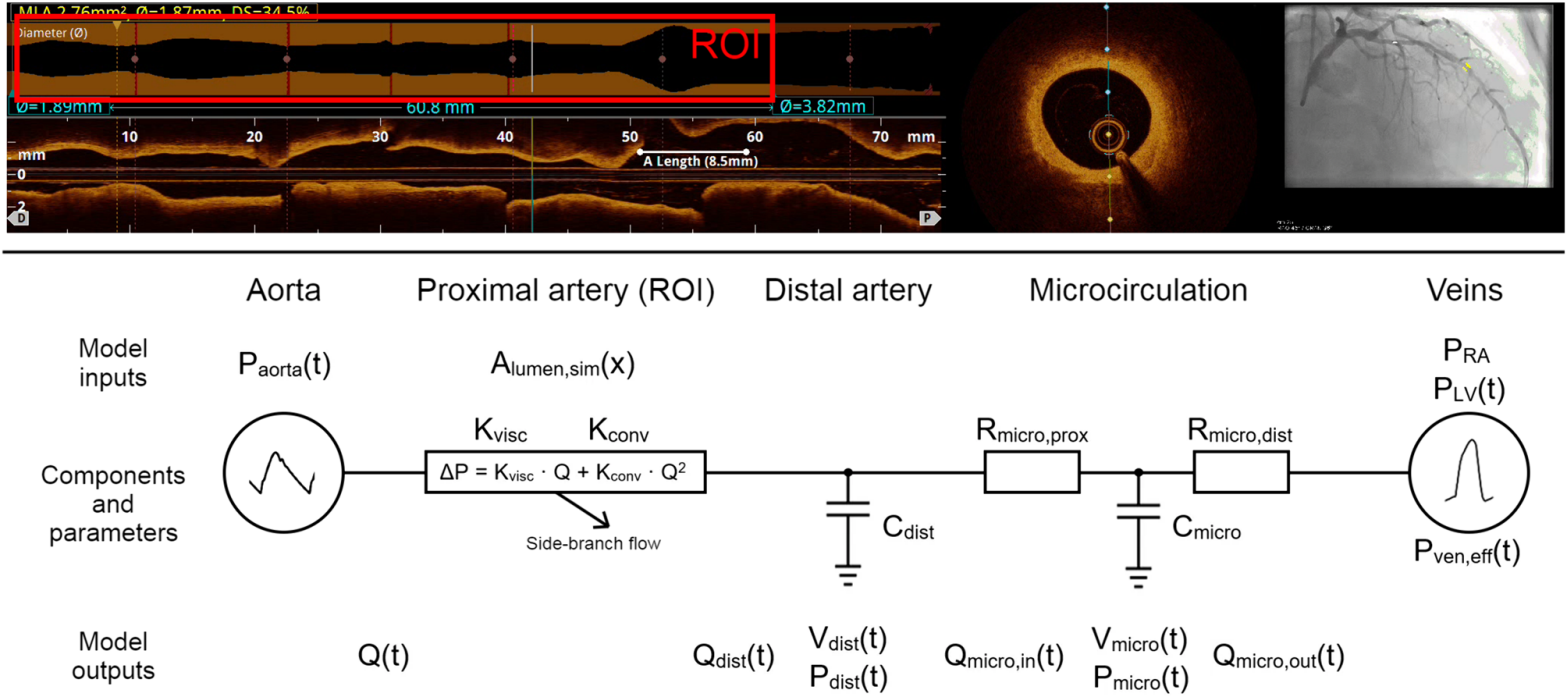
Diagram of simulation model with example of OCT input. Top: Example of OCT image date used to export lumen profile from the region of interest (ROI, red box). Bottom: Diagram of the simulation model. After application of empirical corrections, ROI luminal areal profile is used in the model as A_lumen,sim_(x). See Supplemental Material for model variable definitions

Aortic pressure and 1-dimensional epicardial coronary morphology (ROI lumen profile) were measured in the individual patient and used as inputs to the model, see Fig. 1. In each patient, a proximal and a distal reference lumen area were manually entered based on an inspection of the observed lumen profile. The reference lumen areas were chosen so that the calculated tapered decrease in reference lumen area from the beginning to the end of the ROI corresponded well to the observed lumen area at the least diseased proximal and distal parts of the ROI (see Supplemental Material).

When the simulation model is run, coronary flow and coronary pressure distal to the stenosis are the main outputs, which can then be compared to measurements. Figure 1, lower panel, shows the design of the model. Supplemental Fig. S1 shows a screenshot of the JSim software running the simulation model. A detailed description is provided in the Supplemental Material.

### Calibration of coronary flow

To calibrate the model microvascular resistance with observed values of flow, a reference conductance parameter was determined, representing the microvascular conductance expected downstream of a vessel of 10 mm^2^ reference lumen area. To determine conductance for simulation in specific vessels, this conductance was corrected for measured proximal reference size using the Huo-Kassab scaling law [22]. The reference conductance parameter was adjusted to align overall average coronary flow in the separate vessel territories with observed average flow.

### Patient-specific simulation of fractional flow reserve

For the individual simulation of FFR (simFFR_OCT_), only data on the epicardial coronary lumen dimensions from OCT, the heart rate, and the mean aortic pressure were used.

To produce simultaneous LV and aortic pressure curves, we used a full-circulation model that has been published earlier [23] with a minor modification: a proximal aortic component was added to produce a more realistic aortic pressure curve with a dicrotic notch. Standardized model parameters from a control cohort [23] was used, the heart rate was adjusted to the observed individual value during hyperemia, and peripheral total vascular resistance was adjusted for simulated mean aortic pressure to match observed mean aortic pressure during hyperemia.

The aortic and the LV pressure curves were used as input to the coronary model, and the right atrial pressure was set to 2 mmHg. The mean simulated coronary pressure distal to the ROI (simulated Pd) was calculated, and the simulated hyperemic ratio of Pd/Pa reported as the simFFR_OCT_.

### Adjustment of empirical parameters

The simulations were repeated with different values of the empirical parameter k_lumencorr_, which is a correction factor of the effective lumen radius. This parameter was adjusted to the value resulting in the slope closest to 1 when plotting observed versus simulated distal pressures in the entire cohort.

### Sensitivity analysis

See Supplemental Table S3 for description and result of sensitivity analysis of model parameters.

### Statistics

Groups were compared using Student’s t-test. Correlations were reported as Pearson’s r and agreement between observed and simulated FFR was quantified with Bland–Altman analysis. Sensitivity, specificity, and predictive value was calculated with exact binomial confidence intervals. Diagnostic accuracy was compared between simFFR_OCT_ and percentage area stenosis or plaque burden by ROC analysis and comparison of areas under the curve with DeLong’s test. We used a two-sided significance level of p < 0.05.

All statistical calculations were performed using R for Windows version 4.2.2 with libraries ‘stats’, ‘epiR’, ‘BlandAltmanLeh’, ‘pROC’, ‘ggplot’, and ‘ggbeeswarm’.

### Ethics approval

All patients provided written informed consent before the procedures. The study was approved by The Regional Ethics Committee for the Capital Region of Denmark (protocol number H-17001510).

## Results

### Population and lesion characteristics

In total, 41 patients with stable angina pectoris and coronary artery disease were included in the study, patient characteristics are listed in Supplemental Table S1. Mean age was 65 ± 9 years, 68% were male, and 73% were in Canadian Cardiovascular Society class 2 or worse. LVEF was 57 ± 6%. Right atrial pressure was 4.4 ± 2.5 mmHg (n = 20). 48 coronary vessels were evaluated. None of the included vessels had angiographically visible myocardial bridging. Table 1 lists the lesion characteristics based on angiography, intracoronary pressure and flow measurements, and intravascular imaging with OCT. FFR of the lesions ranged from 0.40 to 0.97, and FFR was ≤ 0.80 in 24 lesions. See Supplemental Table S2 for additional measurements.

**Table 1:** Lesion characteristics with simulation parameters and output

Lesion and model characteristics	n = 48
Coronary artery—LAD/Cx/RCA, n	28 (58%)/13 (27%)/7 (15%)
Intracoronary physiology	
Pa(hyperemia), mmHg	83 (14)
Pd(hyperemia), mmHg	66 (16)
FFR	0.79 (0.14)
Q(hyperemia), mL/s*	2.58 (1.51)**
R(hyperemia), mmHg/(mL/s)*	36.9 (35.4)**
Optical coherence tomography	
Reference vessel area, mm^2^	10.8 (4.5)
Minimal lumen area, mm^2^	2.62 (1.61)
Area stenosis, %	49 (28)
Diameter stenosis, %	65 (26)
Plaque burden, %	76 (11)
Manual model input	
Simulated arterial length (ROI), mm	50 (13)
Proximal reference lumen area, mm^2^	10.57 (2.68)
Distal reference lumen area, mm^2^	5.69 (2.09)
Calculated model input	
K_visc_, mmHg∙s/mL	3.42 (5.55)
K_conv_, mmHg∙s^2^/mL	3.41 (4.73)
C_dist_, µL/mmHg	1.1 (0.4)
C_micro_, µL/mmHg	4.5 (1.7)
R_micro_, mmHg/(mL/s)***	46.2 (33.9)
Simulation outputs	
Simulated Q(hyperemia), mL/s	2.62 (1.10)
Simulated Pd(hyperemia), mmHg	69 (14)
Simulated FFR_OCT_	0.80 (0.12)

*Pa* mean aortic pressure, *Pd* mean distal coronary pressure, *Q* absolute coronary flow, *R* absolute microvascular resistance

Continuous measures reported as mean (SD), categorical as n (%)

*In 1 lesion, Q and R were not available, so n = 47 for these

**In units as reported by the Coroventis software, Q(hyperemia) was 0.155 ± 0.090 L/min, R(hyperemia) was 614 ± 590 mmHg/(L/min)

***The proximal and distal constituents of R_micro_, R_micro,prox_ and R_micro,dist_, were 34.7 ± 25.4 mmHg/(mL/s) and 11.6 ± 8.5 mmHg/(mL/s), respectively

### Coronary microvascular resistance and reference lumen area

Measured hyperemic coronary flow correlated moderately with proximal reference lumen area, r = 0.44, p = 0.002, and coronary flow correlated moderately with microvascular conductance (inverse of microvascular resistance), r = 0.37, p = 0.01. The overall ratio of measured microvascular conductance to proximal reference lumen area was 0.224 ± 0.100 mL/min/mmHg/mm^2^ but differed between left anterior descending artery (LAD) and circumflex artery (Cx)/right coronary artery (RCA). In the LAD, this ratio was 0.255 ± 0.106 mL/min/mmHg/mm^2^, in the Cx/RCA the ratio was 0.182 ± 0.076 mL/min/mmHg/mm^2^ (p = 0.009 for difference).

### Model output and calibration

**Fig. 2:**
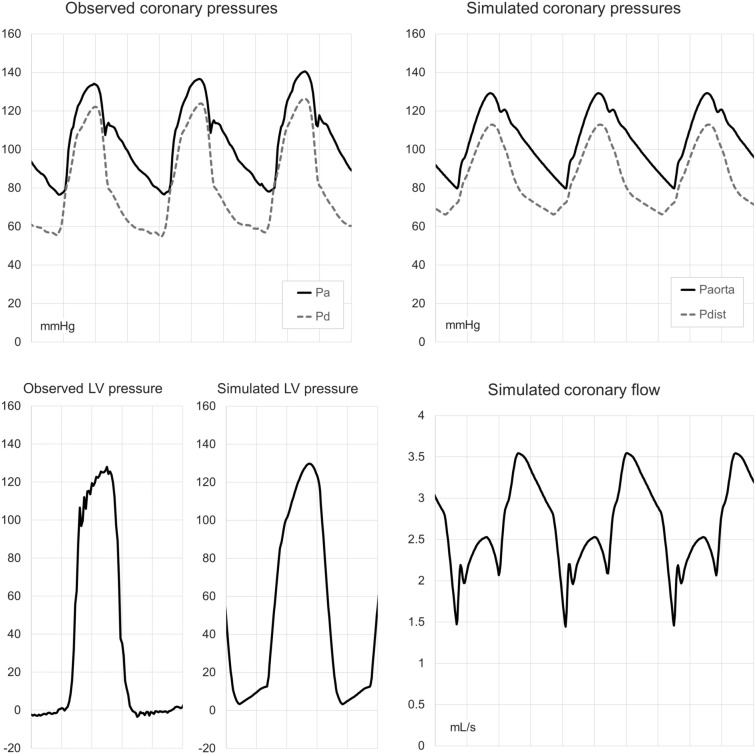
Examples of model output (pressure and flow tracings). Traces are from observed or simulated data in the same patient. Time scale is 200 ms per grid line. Upper left panel: Measured aortic and distal coronary pressure traces. Upper right panel: Simulated aortic and distal coronary pressure traces. Lower left panel: Observed and simulated LV pressure traces. Lower right panel: Simulated instantaneous coronary flow traces, same cycles and phase as upper right panel

Figure 2 shows examples of model output in an individual patient compared with observed values, including simulated whole-cycle coronary flow.

LAD and Cx/RCA arteries were simulated with separate model parameters for the relationship between proximal reference area and microvascular conductance. We achieved the best summary agreement between observed and simulated flow with reference (at a standardized reference lumen size of 10 mm^2^) conductance values of 0.064 mL/s/mmHg for the LAD and 0.043 mL/s/mmHg for the Cx/RCA. See Supplemental Fig. S2 for the agreement between simulated and observed coronary flow in the different arteries after calibration.

The empirical parameter k_lumencorr_ was adjusted to − 0.075 mm to achieve the best slope of the relationship between observed and simulated distal pressure.

See Table 1 for the average simulation model parameters, calculated values, and average model output.

### Predicted versus observed fractional flow reserve

**Fig. 3:**
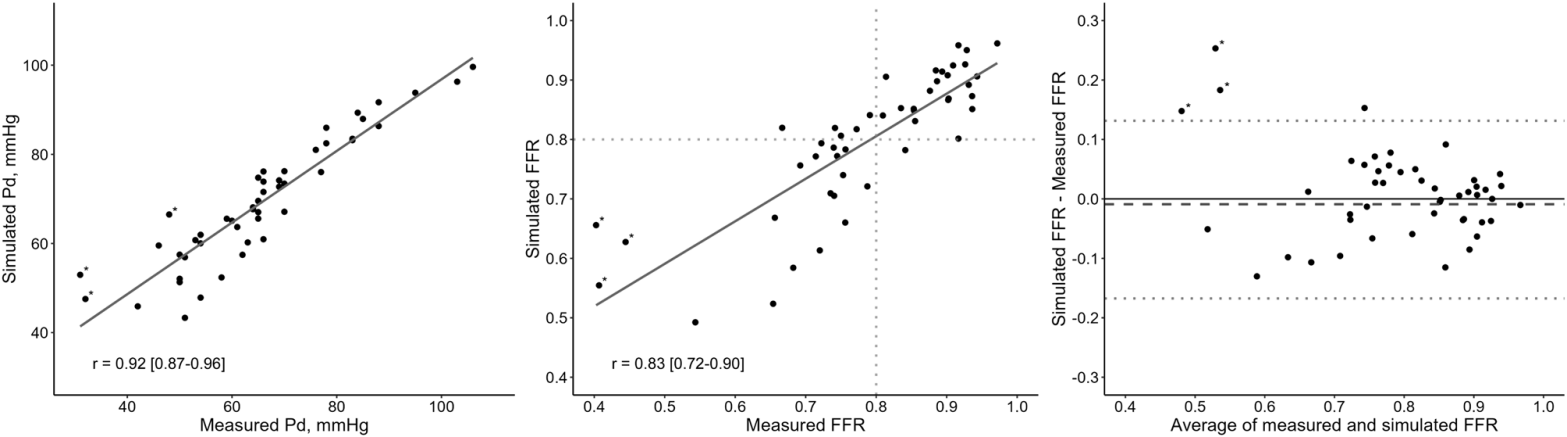
Agreement of simulated and observed values of distal coronary pressure and FFR. Left panel: Measured and simulated distal coronary pressure. r = 0.92 [0.87–0.96]. Middle panel: Measured and simulated fractional flow reserve (FFR). Dotted lines indicate FFR = 0.80. r = 0.83 [0.72–0.90]. Right panel: Bland–Altman diagram with limits of agreement between measured and simulated FFR (± 1.96 SD, dotted lines). Asterixes mark 3 observations with measured FFR < 0.5

Simulated distal coronary pressure and simFFR_OCT_ were strongly correlated with observed values (r = 0.92 [0.87–0.96] for distal coronary pressure and r = 0.83 [0.71–0.90] for FFR), see Fig. 3. The average difference between simFFR_OCT_ and observed FFR was − 0.009 ± 0.076. See Fig. 3 for the Bland–Altman analysis of agreement. The upper and lower limits of agreement were 0.140 and − 0.158, respectively.

The overall diagnostic accuracy for simFFR_OCT_ ≤ 0.80 in predicting observed FFR ≤ 0.80 was 42 out of 48 (0.88 [0.75–0.95]) with a sensitivity of 0.79 (0.58–0.93) and a specificity of 0.96 (0.79–1.00). In this dataset, this represented a positive predictive value of 0.95 (0.75–1.00) and a negative predictive value of 0.82 (0.63–0.94).

In 3 lesions with severe obstruction to flow (measured FFR < 0.5—observations marked with asterixes in Fig. 3), the simulated FFR was overestimated compared with measured FFR, but still clearly hemodynamically significant at a value below 0.7. Excluding these lesions yielded a better numerical agreement with an average difference of 0.003 ± 0.060, with upper and lower limits of agreement are 0.120 and − 0.114, respectively.

The correlation of measured FFR with OCT-derived area stenosis or with OCT-derived plaque burden was moderate (r = − 0.36 [− 0.59 to − 0.07], and r = − 0.47 [− 0.66 to − 0.21], respectively). The AUCs for predicting observed FFR ≤ 0.80 were 0.97 for simFFR_OCT_, 0.43 for OCT derived area stenosis, and 0.63 for OCT-derived plaque burden; the AUC of simFFR_OCT_ was significantly higher than both the others (p < 0.001).

### Sensitivity analysis

See Supplementary Table S3 for sensitivity analysis on selected parameters.

## Discussion

In this study on coronary hemodynamics examining 48 coronary vessels from 41 patients with stable coronary artery disease, we present a new method for estimating FFR based on intracoronary imaging with optical coherence tomography and computer simulation of coronary flow. Based on patient-specific coronary morphology, we simulated whole-cycle realistic pulsatile and total coronary flow. The model performed well in estimating observed FFR, especially in lesions without severe obstruction, and had a diagnostic accuracy comparable to other coronary imaging based FFR estimators.

### Model design

Our approach to modeling pressure loss in coronary arteries is based on the simplified framework of Gould et al. [24], similar to the approach used by others for OCT-based modeling [13]. Apart from the calibration of coronary flow, we used only one empirical constant, a correction of measured lumen diameter, which we expect to be imaging modality specific. Our model includes the effects of pulsatile aortic pressure and the impact of LV contraction on microvascular flow. The addition of these realistic features enhances the interpretability of model parameters such as microvascular resistance, which is thus separated from the effects of LV contraction and systemic pressure, and allows us to simulate absolute total flow correctly. Potentially, this approach could also be used to derive real-time estimates of distal coronary pressure waveforms during invasive examinations.

Side-branch flow was modeled as a continuous leak according to an assumed linear decrease in vessel reference size, which is a simplification of real flow and vessel architecture. The flow just before a large bifurcation will therefore in general be underestimated, and the flow after will be overestimated. An improved prediction might be achieved by modeling the flow distribution at each major bifurcation, preferably by separate modeling of the distal vessel and microvasculature of the side-branch territory.

Only cavity-induced extracellular pressure was included as the mechanism for interaction of myocardial contraction and coronary flow, as this seems the dominant mechanism of a complex interaction [25], even though including shortening-induced pressure somewhat improved the match with experimental data in other studies [25, 26]. To simplify calculations, the interaction was solely modeled though the impact of intramyocardial pressure on the venous side of the microvasculature. In agreement with experimental results [27] and modeling studies [25, 26], interstitial myocardial pressure is assumed to decline linearly from LV cavity pressure at the endocardial surface to zero at the epicardial surface. We modeled the microvascular compartment as one layer, and the intramyocardial pressure felt by the microvasculature is therefore modeled as 50% of LV cavity pressure. To investigate trans-mural differences in the flow of myocardial vessels, a higher-dimension model would be needed [28].

It is an improvement on previous models used to estimate FFR that the current model produces realistic coronary flow, both qualitatively and quantitatively. However, it was unexpected that it was necessary to use different values for calibrating reference myocardial conductance for the LAD versus the Cx/RCA territories. It is known that LAD has the largest flow, but it is not generally assumed that there are different scaling laws for the relationship between size and flow for different arteries [22], even though it is a common clinical experience that stenoses in the LAD are often underestimated visually, while the opposite is true for Cx and RCA [29]. Potential explanations could be differences between the arteries in their tendency to show positive vessel remodeling as an element of generalized atheromatosis, or differences in the severity of diffuse proximal disease, both of which might lead to differences in estimated reference lumen size.

The simulated coronary flow waveform over the cardiac cycle largely matched reported experimental results [26, 27]. Finer details of the flow curve, such as early diastolic flow oscillations, were not reproduced, probably because they arise from wave propagation effects [26] not included in our model.

The tendency of the model to somewhat underestimate the hemodynamic significance of the most severe lesions does not pose a clinical problem because the values still fall in the highly significant territory. A potential reason for the observation is that minimal lumen area in the tightest lesions approaches the cross-sectional area of the OCT catheter. If the OCT catheter has crossed a very tight lesion and keeps it slightly expanded during imaging, the lumen will be overestimated.

### Diagnostic performance

When using OCT to determine hemodynamic significance of a coronary stenosis, a model-based simulated estimate of FFR was here shown to perform better than using the OCT-derived area stenosis based on the MLA. This is in line with earlier studies, as a moderate-to-poor correlation between MLA by intravascular imaging and FFR is a common finding [30].

The correlation of simulated FFR with observed FFR was high, and the diagnostic accuracy of the simulated FFR in establishing hemodynamic significance of a lesion was acceptable at 0.88. Numerical agreement between observed and simulated FFR was only moderate, however, with 95% limits of agreement at ± 0.15. Notably, and clinically relevant, when near-obstructive lesions are excluded, the limits of agreement are better at ± 0.12.

These values are in line with and compare well with the per-vessel accuracy and agreement reported in other imaging-based simulation studies. Using OCT, Huang et al. [16] and Yu et al. [15] reported an accuracy of 0.92 and 0.90, respectively. Limits of agreement were ± 0.14 in the study by Yu. Using intravascular ultrasound data, Bezerra et al. [10] found an accuracy of 0.91, with limits of agreement ± 0.13. With the use of angiography-based reconstruction for modeling flow, Huang et al. [16] found QFR to have an accuracy of 0.87, and limits of agreement at ± 0.14, while Fearon et al. [8] reported an accuracy of 0.92, and limits of agreement at ± 0.14. Estimating FFR from on cardiac computed tomographic angiography image data, Min et al. [4] found a vessel-based sensitivity of 0.80 and a specificity of 0.61, and Nørgaard et al. [5] found FFR-CT to have an accuracy of 0.86, with limits of agreement at ± 0.15.

The results of these studies are remarkably comparable, and it seems simulation-based methods have met a limit to how precisely FFR can be estimated. Based on our data, the residual variation is likely to be caused more by indivicual differences in hyperemic microvascular flow that cannot be estimated from coronary anatomy data alone than by deficiences in simulation methodology. This is suggested by our measurements of absolute flow that showed only a moderate correlation with vessel size. A potential way to improve the models would be to establish which individual clinical features (could be age, hypertension, diabetes, or others) are associated with variations in the relationship between hyperemic flow and vessel size, and include these features in the estimate of hyperemic flow. A larger sample of absolute flow measurements than currently available would be needed for this.

We expect our method to be transferable to other imaging modalities that can produce raw data on vessel dimensions. Estimated vessel dimensions are known to systematically differ between modalities, but it should be possible to overcome this problem by adjusting the empirical k_lumencorr_ parameter of our model.

### Limitations

Several limitations should be noted. For the method to be used clinically, further validation against measured FFR and against clinical endpoints in a larger population and in a multi-center setting would be needed, including evaluation of intra- and inter-observer variability. For this study, the agreement and diagnostic performance were determined in the same data set used for gross calibration, and these results should therefore be interpreted with some caution. In vessels with diffuse disease, positive remodeling, or frank ectasia, this assessment of proximal and distal vessel reference sizes could lead to erroneous results if the estimates do not represent a good approximation of the true non-diseased reference size. Myocardial bridging or inadequate vasodilation may lead to underestimation of the true vessel lumen, and the model can not be expected to perform well in such cases.

## Conclusions

In conclusion, this study introduces a computationally simple simulation model of coronary flow that can be used to simulate pressure loss and FFR over coronary stenoses based on intracoronary optical coherence tomography imaging in individual patients. The model realistically simulates pulsatile, whole-cycle coronary flow, and includes the effect of ventricular contraction on microvascular flow. After calibration, the average simulated coronary flow matched average measured flow. The average difference between simulated FFR and observed FFR was − 0.009 ± 0.076. The overall diagnostic accuracy for simulated FFR ≤ 0.80 in predicting observed FFR ≤ 0.80 was 0.88 (0.75–0.95) with a sensitivity of 0.79 (0.58–0.93) and a specificity of 0.96 (0.79–1.00).

## Impact on daily practice

Simulation of coronary flow based on morphological input can predict hemodynamic significance of stenosis, but most available methods are proprietary, do not include myocardial-microvascular interaction, and are not calibrated to absolute flow. We introduce a new and simple method for simulating coronary flow and pressure based on intracoronary OCT-imaging that realistically simulates whole-cycle pulsatile flow and pressure with side branch flow loss and includes myocardial-vessel interaction. Simulated FFR with the proposed model has moderate agreement with observed values and diagnostic accuracy that compares well with other studies. OCT-based FFR could have clinical utility in allowing simultaneous assessment of coronary morphology and physiology to improve decision making before revascularization and during PCI.

## Supplementary Information

Below is the link to the electronic supplementary material.

Supplementary file1 (DOCX 268 kb)

## Abbreviations

Cx: Circumflex artery
FFR: Fractional flow reserve
simFFR_OCT_: Simulated FFR based on OCT data
LAD: Left anterior descending artery
LV: Left ventricular/left ventricle
OCT: Optical coherence tomography
RCA: Right coronary artery
ROI: Region of interest
