# Supplementary material for: Simulation of coronary fractional flow reserve and whole-cycle flow based on optical coherence tomography in individual patients with coronary artery disease
Source: Int J Cardiovasc Imaging. 2024 Jun 16;40(8):1661–70. doi: 10.1007/s10554-024-03151-6 (PMC11401778; doi:10.1007/s10554-024-03151-6)
Supplement: Supplementary file 1 — Supplementary file1 (DOCX 268 kb) [file 10554_2024_3151_MOESM1_ESM.docx]

Supplemental material

For the manuscript “Simulation of coronary fractional flow reserve and whole-cycle flow based on optical coherence tomography in individual patients with coronary artery disease” by Niels Thue Olsen and Kaining Sheng

Contents

[Model description 2](#_Toc153439316)

[Supplemental tables 6](#_Toc153439317)

[Supplemental figures and figure legends 11](#_Toc153439318)

[Coronary model JSim code 12](#_Toc153439319)

## Model description

Detailed description of the simulation model. See Figure 1 in main article for a diagram of the model.

#### Region of interest (ROI)

The stenotic part of the epicardial coronary artery measured with OCT, the region of interest (ROI), is modeled as a series of short tube segments with a measured lumen area [and a maximal and minimal diameter]. For the simulation, the observed area is corrected with the following formula:

$$A_{lumen, sim}(x)=A_{lumen, obs}(x)+k_{wirecorr}+k_{lumencorr} \cdot2\pi\cdot\sqrt{{A_{lumen,obs}(x)}/\pi}$$

where A_lumen,sim_(x) is the corrected lumen area used for the simulation at position x along the ROI in the direction from proximal to distal, A_lumen,obs_(x) is the observed lumen area from OCT, k_wirecorr_ is a correction factor to account for the cross-sectional area of the coronary pressure wire during measurements (set to -0.1 mm^2^), and k_lumencorr_ is an empirical correction factor of the lumen radius.

A proximal (A_lumen,proximalref_) and a distal reference lumen area (A_lumen,distalref_) is manually selected based on the most healthy segments of the coronary artery, and a reference area, A_lumen,ref_(x), is defined for the entire length of the ROI, based on a linear decrease in reference area from the proximal to the distal end.

#### Side-branch flow

The reduction in flow through the ROI due to side-branch flow is modeled to be related to the decrease in reference lumen area compared to proximal reference lumen area in accordance with the Huo-Kassab scaling law, so that

$$Q_{rel}(x)=\left( \frac{A_{lumen,ref}(x)}{A_{lumen,proximalref}} \right)^{\frac{7}{6}}$$

where Q_rel_(x) is the ratio of coronary flow at the present position of the ROI to coronary flow at the ROI inlet.

#### ROI resistance and flow

The resistance to flow is modeled to be composed of two components: 1) A viscous resistance proportional to flow, and 2) a non-linear component caused by flow convergence, proportional to quadratic flow. The model assumes no pressure recovery in the flow divergence zone.

The relation between flow and pressure difference along the ROI is expressed as

$$\Delta P=K_{visc}\cdot Q+K_{conv}\cdot Q^{2}$$

where Q is the coronary flow at the ROI inlet, and ∆P is the pressure difference between the proximal end (assumed equal to aortic pressure) and the distal end of the ROI.

K_visc_, a coefficient representing viscous resistance of the entire ROI corrected for side-branch flow, is calculated according to the Hagen-Poiseuille equation as

$$K_{visc}=\sum_{x=0}^{ROI length} \left( \frac{8\pi\mu}{{A_{lumen,sim}(x)}^{2}} \right)\cdot Q_{rel}\cdot\Delta x$$

where µ is dynamic viscosity of blood (set to 4 mPa∙s).

K_conv_, the coefficient representing flow convergence losses for the entire ROI, is calculated as

$$K_{conv}=\sum_{x=0}^{ROI length} \left\{ \begin{matrix} \left( \frac{{Q_{rel}(x)}^{2}}{{A_{lumen,sim}(x)}^{2}}-\frac{{Q_{rel}(x-\Delta x)}^{2}}{{A_{lumen,sim}(x-\Delta x)}^{2}} \right)\cdot\frac{\rho}{2}\cdot K_{t}, & acc>0 \\ 0, & acc\leq0 \end{matrix} \right.$$

where acc is the difference between the relative change in A_lumen,sim_ and the relative change in A_lumen,ref_ (where this is positive, flow accelerates along the ROI). ρ is the density of blood (1.06 g∙cm^‑3^). K_t_ is an empirical constant traditionally added to the equation, here set to 1.

The flow equation can be rearranged as

$$K_{conv}\cdot Q^{2}+K_{visc}\cdot Q-\Delta P=0$$

When only forward flow is considered, Q can thus be calculated as the positive root of a second-degree polynomial:

$$Q=\left\{ \begin{matrix} \left( K_{visc}-\sqrt{{K_{visc}}^{2}+4\cdot K_{conv}\cdot\Delta P} \right)/\left( -2\cdot K_{conv} \right), & \Delta P>0 \\ 0, & \Delta P\leq0 \end{matrix} \right.$$

Flow at the distal end of the ROI into the distal epicardial vasculature is calculated as

$$Q_{dist}=Q\cdot\left( \frac{A_{lumen,distalref}}{A_{lumen,proximalref}} \right)^{\frac{7}{6}}$$

The distal epicardial arteries are modeled as a windkessel component with constant, linear compliance and time-varying pressure and volume: P_dist_(t) = C_dist_ · V_dist_(t), where P_dist_ is pressure, C_dist_ is compliance and V_dist_ is volume. C_dist_ is scaled to the reference size of the distal part of the epicardial artery: C_dist_ = A_lumen,distalref_ · 0.2 µL·mmHg^‑1^·mm^‑2^.

The microvasculature is modeled in the same way: P_micro_(t) = C_micro_ · V_micro_(t), where P_micro_ is pressure, C_micro_ is compliance and V_micro_ is volume. C_micro_ is calculated based on the distal reference area of the ROI: C_micro_ = A_lumen,distalref_ · 0.8 µL·mmHg^‑1^·mm^‑2^.

Microvascular resistance distal to the ROI is calculated based on an empirical relationship between artery reference size and microvascular conductance:

$$R_{micro}=\frac{1}{G_{10}\cdot\left( {A_{lumen,distalref}}/{10 {mm}^{2}} \right)^{\frac{7}{6}}}$$

where G_10_ is the empirical microvascular conductance (1/resistance) at a reference vessel area of 10 mm^2^.

Microvascular resistance is divided into two components in series, a proximal component R_micro,prox_ (75% of R_micro_) and a distal component R_micro,dist_ (25% of R_micro_).

Flow from the distal epicardial vasculature into the microvasculature is calculated as

$$Q_{micro,in}={(P_{dist}-P_{micro})}/{R_{micro,prox}}$$

and flow out of the microvasculature into the coronary venous compartment is calculated as

$$Q_{micro,out}={(P_{micro}-P_{ven,eff})}/{R_{micro,dist}}$$

where P_ven,eff_ is effective coronary venous pressure.

#### Effective venous pressure and myocardial-vessel interaction

To account for the effect of myocardial contraction and the change in LV cavity pressure on coronary flow, effective coronary venous pressure with respect to intramyocardial pressure is calculated as the highest value of either the instantaneous average intramyocardial pressure or the right atrial pressure:

$$P_{ven,eff}=\left\{ \begin{matrix} P_{LV}\cdot LVPratio, & P_{LV}\cdot LVPratio>P_{RA} \\ P_{RA}, & P_{LV}\cdot LVPratio\leq P_{RA} \end{matrix} \right.$$

The average instantaneous intramyocardial pressure is calculated as a constant fraction (LVPratio) of LV cavity pressure, for all calculations this constant was set at 0.5 assuming an even distribution of microvascular vessels and a linear decline in intramyocardial pressure through the LV wall from the endocardial to the epicardial surface. P_RA_ was set to 2 mmHg.

## Supplemental tables

**Supplemental Table S1. Patient characteristics**

| **Characteristic** | **n = 41** |
| --- | --- |
| Age | 65.4 (8.7) |
| Male, n (%) | 28 (68%) |
| Height, cm (SD) | 175 (9) |
| Weight, kg (SD) | 88 (15) |
| BMI, kg/m^2^ (SD) | 28.6 (4.0) |
| Smoking, current or previous, n (%) | 27 (66%) |
| Hypertension, n (%) | 26 (63%) |
| Dyslipidemia, n (%) | 30 (73%) |
| Diabetes, n (%) | 5 (12%) |
| COPD, n (%) | 4 (9.8%) |
| Atrial fibrillation*, n (%) | 3 (7.3%) |
| Chronic kidney disease, n (%) | 0 (0%) |
| Peripheral arterial disease, n (%) | 2 (4.9%) |
| Previous MI, n (%) | 4 (9.8%) |
| Previous PCI, n (%) | 8 (20%) |
| Previous CABG, n (%) | 1 (2.4%) |
| CCS class ≥ 2, n (%) | 30 (73%) |
| NYHA class ≥ 2, n (%) | 16 (39%) |
| Systolic BP, mmHg (SD) | 143 (20) |
| Diastolic BP, mmHg (SD) | 81 (11) |
| HR, min^-1^ (SD) | 68 (12) |
| LVEF, % (SD) | 56.5 (6.4) |
| Creatinine, µmol/L (SD) | 73 (13) |
| Medications |  |
| Aspirin, n (%) | 33 (80%) |
| P2Y12-blocker, n (%) | 6 (15%) |
| OAC, n (%) | 3 (7.3%) |
| Statin, n (%) | 39 (95%) |
| Beta blocker, n (%) | 19 (46%) |
| Long-acting nitrate, n (%) | 10 (24%) |
| Calcium antagonist, n (%) | 12 (29%) |
| ACE inhibitor, n (%) | 10 (24%) |
| ARB, n (%) | 9 (22%) |
| Oral antidiabetic, n (%) | 4 (9.8%) |
| Insulin, n (%) | 2 (4.9%) |

BMI: Body mass index, COPD: Chronic obstructive pulmonary disease, MI: Myocardial infarction, PCI: Percutaneous coronary intervention, CABG: Coronary artery bypass grafting, CCS: Canadian Cardiovascular Society, NYHA: New York Heart Association, BP: Blood pressure, HR: Heart rate, LVEF: Left ventricular ejection fraction, OAC: Oral anticoagulant, ARB: Angiotensin II receptor blocker

* Atrial fibrillation was paroxysmal in all cases and not present at time of examination

**Supplemental Table S2. Detailed lesion characteristics**

| **Lesion Characteristics** | **n = 48** |
| --- | --- |
| Coronary artery, n |  |
| LAD | 28 (58%) |
| Cx | 13 (27%) |
| RCA | 7 (15%) |
| Angiographic diameter stenosis, % | 53 (14) |
| Intracoronary physiology |  |
| Pa(rest), mmHg | 88 (12) |
| Pd(rest), mmHg | 81 (13) |
| Pd/Pa(rest) | 0.93 (0.08) |
| RFR | 0.90 (0.13) |
| Pa(hyperemia), mmHg | 83 (14) |
| Pd(hyperemia), mmHg | 66 (16) |
| FFR | 0.79 (0.14) |
| CFR * | 4.35 (3.21) |
| IMR, mmHg∙s * | 20 (10) |
| Q(hyperemia), mL/s * | 2.58 (1.51) ** |
| R(hyperemia), mmHg/(mL/s) * | 36.9 (35.4) ** |
| Optical coherence tomography |  |
| Reference vessel area, mm^2^ | 10.8 (4.5) |
| Minimal lumen area, mm^2^ | 2.62 (1.61) |
| Area stenosis, % | 49 (28) |
| Diameter stenosis, % | 65 (26) |
| Plaque burden, % | 76 (11) |

Continuous measures reported as mean (SD), categorical as n (%). * In 1 lesion, CFR, IMR, Q and R was not available, so n = 47 for these. ** In units as reported by the Coroventis software, Q(hyperemia) was 0.155 ± 0.090 L/min, R(hyperemia) was 614 ± 590 mmHg/(L/min).

**Supplemental Table S3. Model parameters with sensitivity analysis**

| **Model Parameter** | **Value** | **Relative Sensitivity of Model Output (FFR)** |
| --- | --- | --- |
| Input stenosis parameters |  |  |
| Proximal reference lumen area | 10 mm^2^ | -0.205 |
| Distal reference lumen area | 5 mm^2^ | -0.169 |
| K_visc_ | 2.68 mmHg∙s∙mL^‑1^ | - |
| K_conv_ | 2.81 mmHg∙s^2^∙mL^‑2^ | - |
| Fixed parameters |  |  |
| Blood density | 1060 kg/m3 | -0.134 |
| Blood viscosity | 4 mPa∙s | -0.052 |
| k_wirecorr_ | -0.1 mm2 | -0.029 |
| k_lumencorr_ | -0.075 mm | -0.100 |
| K_t_ | 1 | -0.134 |
| Scaling factor for distal epicardial artery compliance | 0.2 µL·mmHg^‑1^·mm^‑2^ | 0.002 |
| Scaling factor for distal microvascular compliance | 0.8 µL·mmHg^‑1^·mm^‑2^ | -0.0003 |
| G_10_ (LAD) | 0.064 mL·mmHg^-1^·s^-1^ | -0.321 |
| Ratio of proximal to distal microvascular resistance | 0.75 | 0.001 |
| LV cavity pressure microvascular compression ratio | 0.5 | 0.184 |
| Bifurcation factor | 7/6 | 0.139 |
| RA pressure | 2 mmHg | 0.002 |

For sensitivity analysis, simulations were run with the input parameters of an individual lesion with intermediate severity (FFR = 0.79). The sensitivity of simulated FFR was now tested for the relevant parameters by increasing each parameter value at a time by 1%. The proportional change in FFR divided by the proportional change in parameter value is reported as relative sensitivity.

The model output evaluated as simulated FFR was moderately sensitive to the manually entered input parameters of reference vessel size proximally and distally, to empirical constant K_t_, to empirical lumen correction constant k_lumencorr_, and to LV cavity pressure microvascular compression ratio (relative sensitivities between 0.10 and 0.21). The model output was highly sensitive to the conductance ratio G_10_ relating microvascular resistance to reference vessel size (relative sensitivity 0.32).

## Supplemental figures and figure legends

**Supplemental Figure S1. Screenshot of JSim platform running the coronary model.**


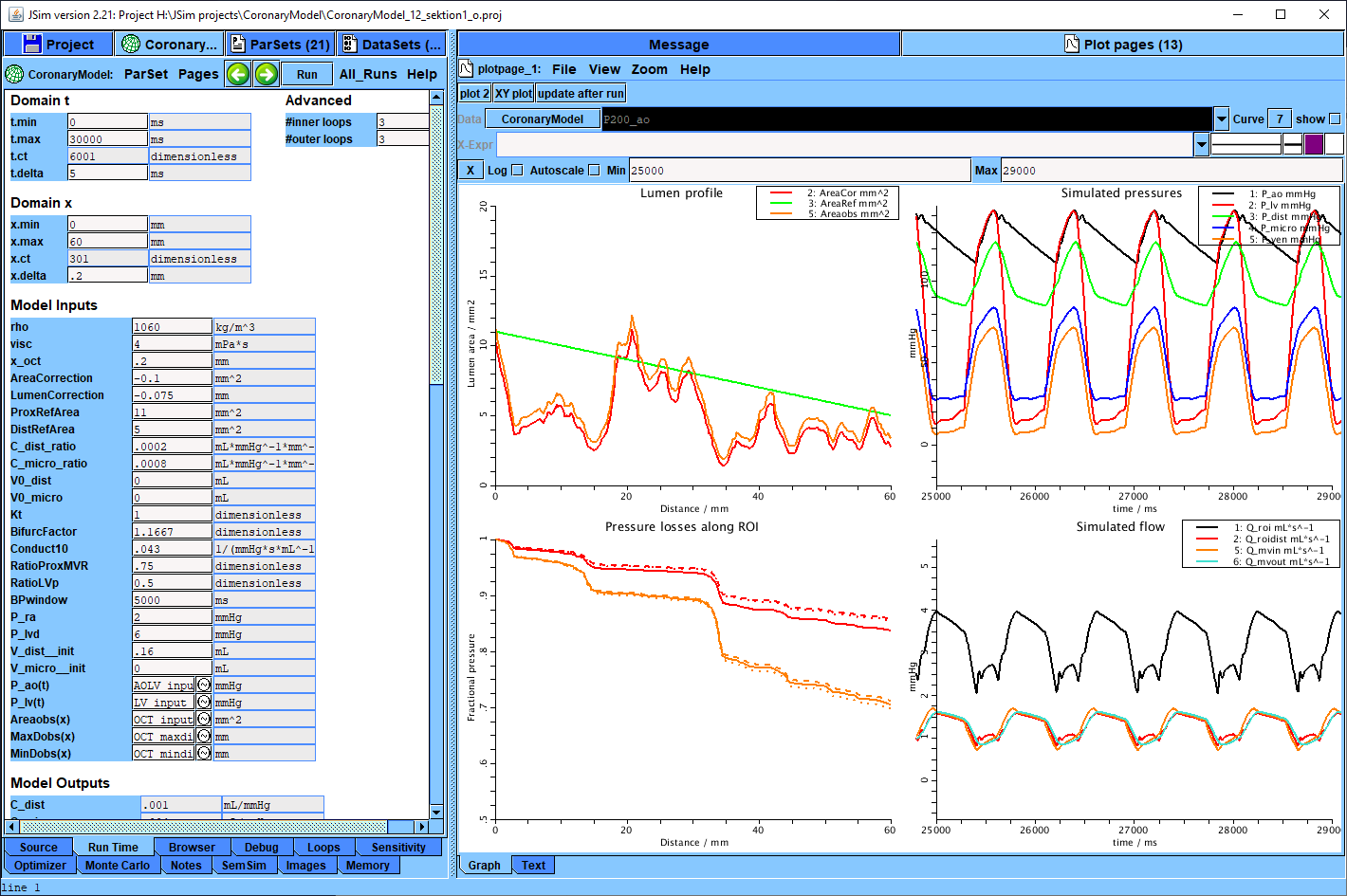


To the right in the window are shown a selection of the possible graphical model outputs. Top left: The lumen profile and the manually entered reference lumen area (green). Top right: Simulated pressures. Bottom left: Pressure loss along the length of the coronary artery for different phases of the cardiac cycle – largest losses are for diastolic phases (orange lines). Bottom right: Simulated coronary flows.

**Supplemental Figure S2. Calibration of simulated average coronary flow with observed values.**


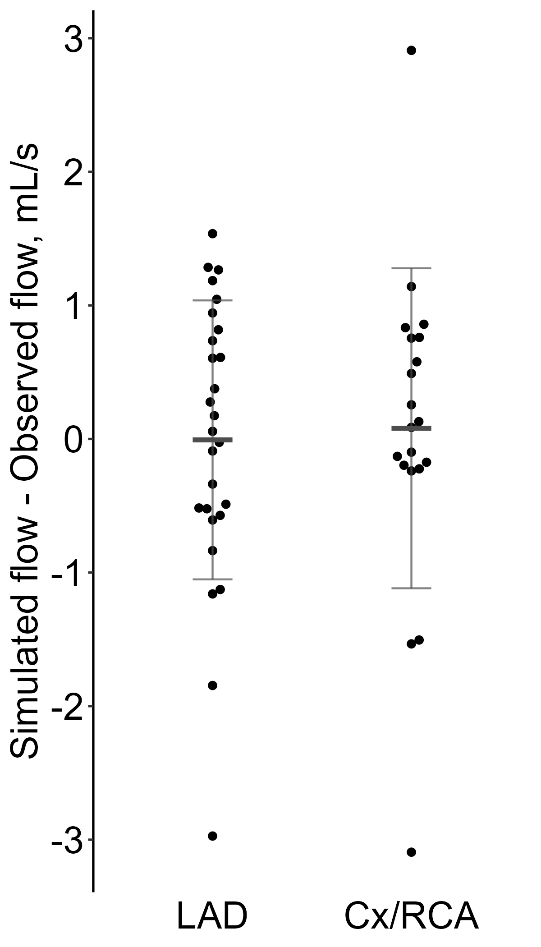


Agreement is shown after calibrating reference conductance separately for LAD and for Cx/RCA. Data for 47 lesions are shown (in one LAD-lesion, absolute flow measurement was not available). Whisker plots are mean difference ± 1 SD.

## Coronary model JSim code

import nsrunit;

unit conversion on;

unit mN = 0.001 N;

unit Pa = 1 N/m^2;

unit mPa = 0.001 Pa;

unit kPa = 1000 Pa;

unit mm = 0.001 m;

unit min = 60000 ms;

math coronary_model{

realDomain t ms;

t.min=0; t.max=100000; t.delta=10;

realDomain x mm;

x.min=0; x.max=75; x.delta=0.2;

real // PARAMETERS

// GLOBAL PARAMETERS

rho = 1060 kg/m^3, // Density of blood

visc = 4 mPa*s, // Dynamic viscosity of blood

x_oct = 0.2 mm, // OCT distance btw points

AreaCorrection = -0.1 mm^2, // Correction of measured area - to correct for wire e.g.

LumenCorrection = -0.075 mm, // Correction of measured lumen radius - to correct for modality-specific differences and obtain effective lumen area

// Manual settings for prox. and dist ref. areas

ProxRefArea = 8 mm^2, // Proximal lumen reference (for manual input)

DistRefArea = 4 mm^2, // Distal lumen reference (for manual input)

// Compliances

C_dist_ratio = 0.0002 mL*mmHg^(-1)*mm^(-2), // Compliance of distal coronary arteries per mm2 reference area

C_micro_ratio = 0.0008 mL*mmHg^(-1)*mm^(-2), // Compliance of distal intramyocardial microvasculature per mm2 reference area

C_dist = C_dist_ratio*DistRefArea, // Corrected for ref size of distal artery

C_micro = C_micro_ratio*DistRefArea, // Corrected for ref size of distal artery

// Unstressed volumes

V0_dist = 0 mL, // Unstressed volume of distal coronary arteries

V0_micro = 0 mL, // Unstressed volume of microvasculature

// Empirical coefficients

Kt = 1 dimensionless, // Empirical coefficient for pressure loss due to flow convergence (Adapted from Young et al. 1977)

BifurcFactor = 1.1667 dimensionless, // Exponential factor to area to obtain proportionality with flow (for Huo-Kassab = 7/6)

// Resistances

Conduct10 = 0.064 1/(mmHg*s*mL^(-1)), // Empirical microvascular conductance (1/R) at an epicardial reference area of 10 mm2

R_micrototal = 1/(Conduct10*(ProxRefArea/(10 mm^2))^BifurcFactor), // Microvascular resistance (arterioles, capillaries, venules) for entire vascular bed distal of proximal ROI

R_micro = R_micrototal/((DistRefArea/ProxRefArea)^BifurcFactor), // Microvascular resistance (arterioles, capillaries, venules) from vascular bed distal to distal ROI

RatioProxMVR = 0.75, // Ratio of arteriole + ½ capillary resistance compared to venular + ½ capillary (Munneke 2022)

R_microprox = R_micro*RatioProxMVR, // The resistance modeled to be proximal to the microvascular capacitor

R_microdist = R_micro*(1-RatioProxMVR), // - distal to the capacitor.

// Parameters for estimation of LV pressure effect on microvasculature

RatioLVp = 0.5 dimensionless, // Ratio for transmission of LV cavity pressure to microcirc

// Timer settings

BPwindow = 3000 ms; // Window for measuring BP

//

private real

HRconverter = 60000 ms/min;

// Initial conditions

// -----------------------------------------------------------------------------

// VARIABLES

// -----------------------------------------------------------------------------

// VARIABLES

extern real

// Pressure input

P_ao(t) mmHg, // Measured aortacurve

P_lv(t) mmHg, // Measured LV pressure

P_ra mmHg, // Right atrial pressure

// Morphology input

Areaobs(x) mm^2, // Measured area

MaxDobs(x) mm, // Measured max diameter

MinDobs(x) mm; // Measured min diameter

real

// Morphology

AreaCor(x) mm^2, // Effective coronary area

AreaChange(x) mm^2, // Area change

AreaRef(x) mm^2, // Reference area at point x

Q_rel(x) dimensionless, // Flow at x compared to flow at entry after correcting for sidebranch loss

// Pressures

P_dist(t) mmHg, // Pressure of distal epicardial arteries

P_micro(t) mmHg, // Pressure of microvasculature ("mid")

P_ven(t) mmHg, // Coronary venous pressure

P_roi(t,x) mmHg, // Instantaneous pressure along the ROI

// Flows

Q_roi(t) mL*s^(-1), // Coronary flow into proximal ROI

Q_roidist(t) mL*s^(-1), // Coronary flow from ROI to distal arteries

Q_mvin(t) mL*s^(-1), // Coronary flow from distal epi arteries into microvasculature

Q_mvout(t) mL*s^(-1), // Coronary flow from microvasculature into coronary veins

// Volumes

V_dist(t) mL, // Volume of distal epicardial arteries

V_micro(t) mL, // Volume of microvasculature

// Resistance coefficients

R_roi(x) mmHg*s*mL^(-1),

R_roisum mmHg*s*mL^(-1),

R2_roi(x) mmHg*s^2*mL^(-2),

R2_roisum mmHg*s^2*mL^(-2);

// -----------------------------------------------------------------------------

// INITIAL CONDITIONS

// -----------------------------------------------------------------------------

// Initial Conditions

when(t=t.min) {

// Volumes

V_dist = 0;

V_micro = 0;

}

// -----------------------------------------------------------------------------

// SYSTEM OF EQUATIONS

// -----------------------------------------------------------------------------

// Area equations

AreaCor = Areaobs+AreaCorrection+LumenCorrection*PI*2*(Areaobs/PI)^.5; // if correction for hydraulic area add: "* 2 * MaxDobs * MinDobs / (MaxDobs^2+MinDobs^2)"

AreaChange = AreaCor(x) - AreaCor(x-x.delta);

AreaRef = ProxRefArea - (ProxRefArea-DistRefArea)/(x.max-x.min)*x;

Q_rel = (AreaRef/ProxRefArea)^(BifurcFactor);

// ROI Coronary resistance and flow

// Viscous resistance to be multiplied by flow

R_roi = 8*PI*visc/(AreaCor^2)*x.delta // Viscous resistance

* Q_rel ; // Corrected for lower flow more distally due to sidebranch flow

R_roisum = sum(R_roi@x); // Summary coefficient for entire ROI

// Flow convergence non-linear effect to be multiplied by squared flow

// (assuming no pressure recovery)

R2_roi = if (AreaCor(x)/AreaCor(x-x.delta)<Q_rel(x)/Q_rel(x-x.delta)) // Flow is only accelerated if area decreases more than reference area

(Q_rel(x)^2/AreaCor(x)^2-Q_rel(x-x.delta)^2/AreaCor(x-x.delta)^2)*rho/2 // Pressure loss due to convergent acceleration, corrected for lower flow more distally due to sidebranch flow assuming area conservation

* Kt // Empirical coefficient

//

else

0 ;

R2_roisum = sum(R2_roi@x); // Summary coefficient for entire ROI

// Flow is calculated based on pressure difference and the linear and quadratic components

// Only antegrade flow is allowed

// Solved as positive root of a second degree polynomial

// R2*Q^2 + R*Q - dP = 0

Q_roi = if((P_ao-P_dist) > 0)

(R_roisum-sqrt(R_roisum^2+4*R2_roisum*(P_ao-P_dist)))/(-2*R2_roisum)

else

0 ;

Q_roidist = Q_roi*(DistRefArea/ProxRefArea)^(BifurcFactor);

P_roi = P_ao - sum(x = x.min to x, R_roi)*Q_roi - sum(x = x.min to x, R2_roi)*Q_roi^2; // Local pressure along the ROI (monitor)

// Effective distal venule pressure

P_ven = if (P_lv*RatioLVp > P_ra)

P_lv*RatioLVp

else

P_ra;

// Coronary flow

Q_mvin = if ((P_dist-P_micro<0) and (V_micro<=0))

0

else

(P_dist-P_micro)/R_microprox;

Q_mvout = if ((P_micro-P_ven>0) and (V_micro<=0))

0

else

(P_micro-P_ven)/R_microdist;

// Conservation of mass equations

V_dist:t = Q_roidist-Q_mvin;

V_micro:t = Q_mvin-Q_mvout;

// Vessel pressures

P_dist = (V_dist-V0_dist)/C_dist;

P_micro = (V_micro-V0_micro)/C_micro + P_lv*RatioLVp;

} // END OF MML CODE

/*

Original author: Niels Thue Olsen 2023.

Developed for the JSim software platform:

Copyright (C) 1999-2009 University of Washington. From the National Simulation Resource,

Director J. B. Bassingthwaighte, Department of Bioengineering, University of Washington, Seattle WA 98195-5061.

Academic use is unrestricted. Software may be copied so long as this copyright notice is included.

*/
